# Morpheme knowledge is shaped by information available through orthography

**DOI:** 10.3758/s13423-025-02830-2

**Published:** 2025-12-08

**Authors:** Maria Korochkina, Holly Cooper, Marc Brysbaert, Kathleen Rastle

**Affiliations:** 1 Department of Psychology, Royal Holloway, University of London, Egham Hill, Egham, Surrey TW20 0EX UK; 2 Department of Experimental Psychology, University of Ghent, Ghent, Belgium

**Keywords:** Morphology, Reading, Text experience, Natural language processing, Learning

## Abstract

A large portion of words in a language are formed by combining smaller meaningful units called morphemes (e.g., *teach* + *-er* → *teacher*). Understanding a language’s morphology is vital for skilled reading as it allows readers to interpret both familiar and unfamiliar words (e.g., *tweeter*). It is widely agreed that children rely on reading experience to acquire morpheme knowledge in English, and emerging research suggests that different aspects of this experience may impact affix learning in different ways. We contrasted three potential definitions of what constitutes readers’ affix experience using the morpheme interference paradigm with 120 adults. We found that skilled readers’ affix knowledge most closely aligns with a definition proposing that affix learning is primarily supported by experience with words in which affixes are identifiable without specialised linguistic knowledge. Due to the nature of morpheme presentation in English orthography, this excludes a significant number of genuinely complex words, while including affix-like patterns in non-meaningful contexts (e.g., *-er* in *corner*). This definition also posits that these morphological false alarms actively hinder learning. Our research represents a critical step towards a psychologically realistic theory of morpheme learning from text experience.

## Introduction

A hallmark of skilled reading is the ability to compute the meanings of printed words rapidly and without the need to translate symbols of writing back into sounds (for a comprehensive review, see Castles et al., 2018). Research shows that expert readers accomplish this feat because, for a large proportion of words in a language, the relationship between form and meaning is not arbitrary but underpinned by morphology (Rastle, 2019a). Decades of research have documented the remarkable sensitivity of skilled readers to the morphological regularities of their language (for reviews, see Amenta & Crepaldi, 2012; Stevens & Plaut, 2022). The impact of morphology on word recognition varies across languages and writing systems (Frost, 2012). In this article, we focus on the impact of derivational morphology on skilled reading in English and the process by which this knowledge is acquired.

In English, it is well established that skilled readers analyse complex-looking words in terms of their constituent morphemes. For example, printed words are recognised more quickly if they have high-frequency stems (e.g., Taft & Ardasinski, 2006) or belong to large morphological families (e.g., Juhasz & Berkowitz, 2011). Words (e.g., *dark*) are also recognised faster when preceded by a morphologically related (e.g., *darkness*) compared to a morphologically unrelated (e.g., *fullness*) word (for a review, see Rastle & Davis, 2008). Research further shows that words undergo this rapid morphological analysis regardless of their lexical status. For instance, readers find it harder to reject nonwords with morphological structure (e.g., *earist*) compared to those without morphological structure (e.g., *earilt*), a phenomenon known as the morpheme interference effect (e.g., Crepaldi et al., 2010; Taft & Forster, 1975). Together, these studies demonstrate that expert readers routinely use their knowledge of morphemes to interpret both familiar and unfamiliar printed words.

Given this fundamental contribution of morphological knowledge to skilled reading, it is important to understand how the English reading system becomes attuned to the morphological regularities in English writing. Children’s understanding of morphological structure begins through spoken language and early reading experiences (e.g., Carlisle & Kearns, 2017). Although affixes do not occur in isolation, repeated exposure to morphologically complex words helps children recognise that many words are made up of smaller, meaningful parts that often carry consistent meanings (Grainger & Beyersmann, 2017; Schreuder & Baayen, 1995). For example, encountering pairs like *insane* and *sane* or *complete* and *incomplete* may lead them to infer that the element *in-* signals absence or negation. While this awareness is sometimes reinforced through formal literacy instruction, limited time is devoted to explicit and structured teaching of morphology in English-speaking countries. As a result, theoretical accounts suggest that children acquire most of their morphological knowledge through exposure to written text (Rastle, 2019b). What makes this process so intricate is that the *systematicity* with which affix morphemes contribute to word meanings is *graded*: the same affix can have a clear and meaningful contribution in some words (e.g., *-er* in *baker*, *teacher*, and *banker* denotes agency), while in others its contribution to meaning may be more tenuous (e.g., *dresser*) or even absent (e.g., *corner*). These varying degrees of systematicity in how a morpheme’s spelling maps to meaning suggest that accumulated experience with printed words may play a crucial role in shaping both which morphemic regularities are processed and acquired and how this learning unfolds (Gonnerman et al., 2007; Plaut & Gonnerman, 2000; Seidenberg & Gonnerman, 2000).

Empirical work has corroborated this idea. Ulicheva et al. (2020) created nonwords with English suffixes that varied in the consistency with which they indicated grammatical class and tested skilled readers’ sensitivity to this information. To illustrate, the suffix *-ness* indicates noun status more reliably than the suffix *-y*, which also appears in nouns (e.g., *robbery*) but is far more common in adjectives (e.g., *guilty, hungry, witty*). Ulicheva et al. (2020) found that nonwords with the suffix *-ness* (e.g., *rabness*, *tobness*, *juzness*) were more likely to be classified as nouns than adjectives and that the participants’ eyes were more likely to regress back to a nonword when it appeared in an adjective context than in a noun context. Likewise, participants were more likely to use the spelling *-ness* when the spoken form of the nonword ending in [nɪs] occurred in a noun context (e.g., “The lighthouse keeper hated the lonely [mɪbnɪs] of his occupation”) rather than in a verb context (e.g., “The pupils always quietly [dædʒnɪs] when they sit in the back row”) (see also Treiman et al., 2020, for similar findings). Evidence from artificial learning experiments further supports the idea that morphemic knowledge develops in a way that reflects the strength of statistical regularities in print. Tamminen et al. (2015) presented adult readers with definitions of novel words exhibiting an underlying morphological structure (e.g., a familiar stem combined with the novel affix *-lomb*, as in *fetchlomb*, *mowlomb*, *lynchlomb*) and tested their ability to generalise knowledge of the new affix. No instruction on the affix meanings was provided; however, the definitions of the novel words related back to their stems (e.g., *fetch*, *mow*, *lynch*) in a way that allowed participants to infer a potential function of the novel affix. Tamminen et al. (2015) observed that knowledge of the novel affixes was determined by the number of different stems with which the affix appeared and whether it had a consistent, meaningful function across these words.

This body of research leads to the notion that affix learning can be conceptualised as a statistical learning problem, and that the variability in words within which an affix is encountered (i.e., affix *type* frequency) is a key determinant of this learning (for further discussion on the importance of type over token frequency in lexical processing, see De Jong et al., 2000; Hsieh et al., 2024; Schreuder & Baayen, 1997; and on the role of input variability in learning across cognitive domains, see Raviv et al., 2022). However, these theoretical perspectives have not considered the viewpoint of a child who must navigate their reading experience without systematic guidance or instruction on which aspects of their lexical experience to prioritise in learning about morphemes. This is important because morphemic regularities in natural language vary along multiple dimensions that may influence learning. One such dimension is the ease with which affixes can be identified in complex words by a reader without specialised linguistic knowledge, relying almost entirely on orthographic cues for segmentation. To illustrate, the words *include*, *inject*, *insomnia*, and *independent* all contain the prefix *in-*; however, only in *independent* does removing *in-* yield a meaningful English word (*dependent*), making the meaning of *in-* easier to identify in this case. In contrast, although readers may recognise the recurring sequence *in* in the other words and have some tacit sense of its meaning from spoken language experience, it is much harder to infer the precise meaning of this prefix without familiarity with the Latin roots of the bound stems *clude*, *ject*, and *somnia*. This difficulty is further compounded by that fact that, unlike *insomnia*, the words *include* and *inject* do not convey the idea of absence or negation. Critically, a recent analysis of books popular with children aged 7–16 years demonstrates that about half of the prefixed and nearly a third of the suffixed words used in these books contain bound stems (Korochkina & Rastle, 2025). Likewise, linguistic knowledge is required to distinguish useful cases like *teacher* and *independent* from misleading ones like *corner* and *intent* as, orthographically, they can all be segmented into a meaningful stem (*teach*, *dependent*, *corn*, *tent*) and an affix (*in-*, *-er*). Morphological false alarms like *corner* and *intent* are problematic because they may impede affix learning by causing readers to assume false relationships between words and misinterpret the affix’s meaning (Korochkina & Rastle, 2025). These findings suggest that readers’ experience of morphemes may be governed by principles other than those described in linguistic theories of English morphology.

In this article, we ask how different aspects of text experience contribute to readers’ morpheme knowledge. Building on previous research, we assess this knowledge using the morpheme interference paradigm (Crepaldi et al., 2010; Dawson et al., 2018; Taft & Forster, 1975). We examine whether participants’ performance on this task differs based on how individual affixes appear in print by contrasting three hypotheses about what defines a reader’s experience of an affix. Hypothesis 1 is based on a linguistic definition of affixes and measures affix type frequency by counting all occurrences where a complex-looking word is historically formed through derivational affixation, regardless of a reader’s ability to recognise the affixes within these words (dictionary-based type frequency). The psychological basis for this definition is that, given the abundance of words with bound stems in English, readers might still benefit – at least to some extent – from their experience with these words, even if their morphological structure is not immediately apparent. Hypotheses 2 and 3 propose that readers can only draw on experience with words where affixes are easily identifiable. This includes genuinely complex words whose stems can function as standalone words (e.g., *independent* but not *inject*) as well as false alarms (e.g., *intent*), which can be segmented into morphemes based on their spelling (orthography-based type frequency). Hypothesis 3 further posits that false alarms should incur a learning penalty, as their presence may hinder learning by introducing uncertainty about the function of a given affix in complex-looking words.

## Method

Following previous research, we hypothesised that adult readers would make more errors and take longer to reject morphologically structured nonwords (e.g., *woodness*) compared to their orthographic controls (e.g., *woodnels*), reflecting their acquired sensitivity to morphological structure. We designed the morphologically structured nonwords using affixes that were reasonably common but differed in how often they were detectable in complex words and in their involvement in false alarms, based on a corpus of 1,200 books popular with British children and young people (Korochkina & Rastle, 2025; Korochkina et al., 2024). We anticipated that accuracy and response times to these morphologically structured nonwords would depend on the quantity and quality of affix exposure in print. Specifically, nonwords containing affixes with higher frequency, greater detectability, and fewer false alarms were expected to be harder to reject (i.e., rejected less accurately and more slowly) than those including affixes with lower frequency, lower detectability, and more false alarms. To identify which of our three definitions of affix exposure (dictionary-based type frequency, orthography-based type frequency, and orthography-based type frequency adjusting for the false alarm penalty) best explained participants’ performance, we built separate models for each and then compared the fit of these models. Below, we outline the stimulus design and experimental procedure and describe how the relevant measures were computed. This experiment was not pre-registered. Stimuli, data, and analysis code for this project are available on the Open Science Framework (https://osf.io/yq9h7/).

### Participants

Participants were 120 adults recruited via Prolific (mean age = 30 years, *SD* = 6.57; 63 female, 56 male, one non-binary). Participants were based in the UK, reported English as their first language, and had no diagnosed language disorders. Three additional participants were tested but removed due to a programming error (2) and lack of engagement with the task (1). The sample size was determined by the need to maximise the number of observations per participant per affix, as well as the number of distinct affixes and stems paired to create nonwords, while keeping the experiment short enough to prevent fatigue. We therefore settled on 12 affixes and 120 distinct stems, with each participant seeing each affix paired with ten different stems from the stem pool. This resulted in 1,440 possible stem-affix pairs. To ensure that each pair was seen an equal number of times, we required 120 participants. Brysbaert and Stevens (2018) recommend at least 1,600 observations per condition in a factorial design. Our study far exceeded this, with 14,440 observations per condition (nonwords with vs. without morphological structure) for the accuracy data, and 13,834 (no morphological structure) and 11,387 (morphological structure) observations for the response-time data (correct ‘no’ responses only). Participants were paid £3 for completing the study. The experiment received ethics approval from the Research Ethics Committee at Royal Holloway, University of London (Ref. 322).

### Materials

One hundred and twenty morphologically structured nonwords and 120 control nonwords without morphological structure were created. Six prefixes (*un-*, *mis-*, *dis-*, *pre-*, *re-*, *de-*) and six suffixes (*-ness*, *-ly*, *-able*, *-er*, *-ic*, *-ate*) were selected based on their usage in the CYP-LEX database (Korochkina et al., 2024). These affixes were selected because they had reasonably high dictionary-based type frequency (ensuring that they were familiar to participants) while offering variability in detectability and involvement in false alarms, as reported in Korochkina and Rastle (2025). Korochkina and Rastle (2025) determined the morphological structure of the distinct words in the CYP-LEX corpus using MorphoLex, a lexical database that details the morphological structure of nearly 70,000 English words (Sánchez-Gutiérrez et al., 2018). They calculated the dictionary-based type frequency of each affix by counting the distinct genuinely complex words in which each affix appeared in the CYP-LEX corpus. Affixes that linguists might treat as distinct morphemes based on syntax or etymology but that share the same orthographic form (e.g., *-er* in *bigger* and *teacher*, or *in-* in *inborn* and *include*) were collapsed in these counts, as it is unclear how an average reader would distinguish and reliably categorise them as separate morphemes. The orthography-based type frequency of each affix was computed by summing the number of distinct genuinely complex words in which the affix could be detected without sophisticated etymological knowledge (i.e., the knowledge required to accurately parse genuinely complex words with bound stems), and the number of distinct words that could be mistakenly parsed as containing the affix based on their spelling (i.e., false alarms). Words were classified as false alarms if they could be segmented into an existing English word and an affix, and a potential stem was considered viable if it appeared as a standalone word in the CYP-LEX corpus (Korochkina & Rastle, 2025). We did not incorporate phonology into our frequency computations. While phonological similarity may influence whether a reader assumes a relationship between two words, its contribution is far from straightforward. For example, false alarms and their apparent orthographic stems can vary in their degree of phonological overlap (e.g., *corn* [kɔːn] – *corner* [ˈkɔːnə] vs. *broth* [brɒθ] – *brother* [ˈbrʌðə]). At the same time, genuinely morphologically related pairs also often show considerable phonological divergence (e.g., *signature* [ˈsɪɡnətʃə] – *sign* [saɪn], *magic* [ˈmædʒɪk] – *magician* [məˈdʒɪʃən]). This complexity suggests that phonological factors warrant dedicated investigation, which falls beyond the scope of the present study. All frequency statistics are presented in Table 1.

**Table 1:** Lexical statistics for the 12 affixes used in the morpheme interference task based on the CYP-LEX database (Korochkina et al., 2024)

Affix type	Affix	Dictionary-based type frequency	Orthography-based type frequency (N distinct words in which the affix is easily detectable + N false alarms)	False alarm penalty
Prefix	un-	833	788 (774 + 14)	0.129
	mis-	179	188 (172 + 16)	0.420
	dis-	501	464 (410 + 54)	0.519
	pre-	184	181 (131 + 50)	0.850
	re-	895	880 (597 + 283)	0.906
	de-	413	413 (214 + 199)	0.999
Suffix	-ness	877	879 (876 + 3)	0.033
	-ly	2,345	2,238 (2,205 + 33)	0.111
	-able	699	354 (320 + 34)	0.456
	-er	2,799	2,807 (2,342 + 465)	0.648
	-ate	1,569	408 (340 + 68)	0.650
	-ic	791	297 (222 + 75)	0.815

For each affix, the table reports: (1) dictionary-based type frequency, representing the number (N) of distinct words containing the affix based on etymological information; (2) orthography-based type frequency, calculated as the sum of genuinely complex words in which the affix can be easily detected (first number in parentheses) and false alarms (second number in parentheses); and (3) the false alarm penalty value. The affixes are grouped by type (prefix or suffix) and ordered by increasing false alarm penalty within each group

We quantified the false alarm penalty for each affix using *Shannon entropy* (Shannon, 1948), an information-theoretic measure which has been shown repeatedly to be psychologically relevant (for a recent discussion, see, e.g., Westbury & Yang, 2025,). This measure was computed using the formula from Shannon (1948), adapted to our specific case:

$$H=-\left({p}_{gc} {log}_{2}\left({p}_{gc}\right)+{p}_{fa} {log}_{2}\left({p}_{fa}\right)\right)$$

where ${p}_{gc}$ represents the probability that the letter string associated with a given affix signifies a genuinely complex word, while ${p}_{fa}$ represents the probability that the letter string does not carry a meaningful function and instead signifies a false alarm. These probabilities are calculated as follows:

$${p}_{gc} = \frac{N\ \text{genuinely complex detectable words}}{\text{Orthography-based type frequency}}$$ and $${p}_{fa} = \frac{N\ \text{false alarms}}{\text{Orthography-based type frequency}}$$

The penalty value (*H*) for affixes ranges from 0 to 1. Higher values indicate greater uncertainty about the function of the orthographic pattern associated with an affix in the words that contain it, while lower values reflect lower uncertainty. For example, the suffix *-ness* can be easily identified in all but one of the genuinely complex words containing the orthographic pattern *ness*, and this pattern appears in only three false alarms (see Table 1). Thus, ${p}_{gc}$ for *-ness* is $\frac{876}{879} =0.997$, and ${p}_{fa }= \frac{3}{879} = 0.003$. This results in a penalty value of 0.033, indicating minimal uncertainty about the function of *ness* in words where it occurs. In contrast, the orthographic pattern *ic* is associated with much greater uncertainty: it can be detected in 222 genuinely complex words but also appears in 75 false alarms, resulting in a penalty value of 0.815.

The false alarm penalty values for all affixes used in our study are reported in Table 1, with affixes ordered by increasing penalty within each affix type (prefix or suffix). Note that there is not always a direct correspondence between affix frequency and false alarm penalty. For example, the suffixes *-er* and *-ate* have similar false alarm penalties (0.648 and 0.650, respectively); however, *-er* is much more frequent than *-ate*, suggesting that the false alarm penalty is likely to have a more negative impact on the learning of *-ate* than on that of *-er*. In contrast, the suffix *-ly* is both highly frequent and does not participate in many false alarms.

Each of these 12 affixes was combined with 120 monomorphemic words (e.g., *wood*, *sheep*) to create 1,440 morphologically structured nonwords (e.g., *woodness*, *sheeper*). The monomorphemic words were selected from the CYP-LEX database (Korochkina et al., 2024), ensuring that none ended in letters that often undergo orthographic changes when combined with derivational affixes (e.g., *e*, *y*, as in *steady* + *-ness* → *steadiness*, *advise* + *-able* → *advisable*). The selected monomorphemic words had Zipf frequencies ranging from 2.92 to 5.10 in the 1,200 books of the CYP-LEX database and were four to five letters in length. To ensure that none of the novel morphemic combinations formed existing words, we checked each against the SUBTLEX-UK database (van Heuven et al., 2014) and then manually reviewed them to avoid any orthographic similarities. The construction of orthographic controls (i.e., nonwords without morphological structure) followed Crepaldi et al. (2010) by modifying one letter in the affix of the morphologically structured nonwords. For two-letter affixes, the last letter was replaced (e.g., *-er* → *-el*), while for affixes with three or four letters, the second or third letter was replaced (e.g., *-ate* → *-afe*, *-ness* → *-nels*). These modified non-affixes were then paired with the same monomorphemic words used for the morphologically structured nonwords, yielding a total of 1,440 control nonwords (e.g., *sheepel*, *motorafe*, *woodnels*). All nonwords were between six and nine letters in length and were both orthographically and phonotactically legal in English.

To ensure an equal number of ‘yes’ and ‘no’ responses in the lexical decision task and that the number of words with and without morphological structure among the existing words (for which a ‘yes’ response was expected) was the same as that among the nonwords, we selected 120 multimorphemic and 120 monomorphemic words from the CYP-LEX database. These words were matched to the nonwords in length, Zipf frequency in the CYP-LEX database, and orthographic neighbourhood density as reported in the Clearpond database (Marian et al., 2012). The multimorphemic words included the 12 affixes used in the morphologically structured nonwords, as well as 11 additional affixes (*im-*, *-en*, *-less*, *-ful*, *-al*, *-est*, *-ion*, *-ish*, *-ity*, *-ist*, *-ful*). The full list of the stimuli used in the experiment is available in Appendix A in the Online Supplementary Material (https://osf.io/yq9h7/).

Each participant saw 480 letter strings: 240 nonwords (‘no’ responses expected) and 240 existing words (‘yes’ responses expected). The nonwords included 120 morphologically structured nonwords, with each affix paired with ten different stems, and their 120 non-morphologically structured orthographic controls. The words included 120 morphologically complex and 120 morphologically simple words. All participants saw the same existing words, but the stem-affix pairings in the nonwords differed across participants. This approach aimed to minimise the influence of any idiosyncratic stem-affix combinations (e.g., potential part-of-speech constraints associated with certain affixes; although note that whether readers are sensitive to such patterns is yet to be shown). The order of the letter strings was pseudo-randomised for each participant so that (a) no more than five words or nonwords and (b) no more than two letter strings with the same affix appeared consecutively.

### Procedure

The experiment was created and hosted using Gorilla (https://www.gorilla.sc/). Each trial began with a fixation cross presented in the centre of the screen for 500 ms, which was then replaced by a lowercase letter string (word or nonword; font size 60 px). The letter string remained on the screen until a response was made. Participants were instructed to press the letter ‘A’ on the keyboard if they believed the letter string was a real word, or the letter ‘L’ if they believed it was not a real word. They were asked to respond as quickly and as accurately as possible. The next trial began 1 s after the participant’s response. The task started with six practice trials and included two breaks, each after 160 successive trials. The entire experiment took approximately 20 min to complete.

### Analysis

The data were analysed in R, version 4.2.1 (R Core Team, 2022). Before conducting statistical analyses, we examined the distribution of response times across participants and items. Data points that clearly fell outside the distribution — responses faster than 200 ms or slower than 3,000 ms – were removed, resulting in data loss of less than 0.05%. Data for the existing words (the expected ‘yes’ decisions) were not analysed. We used (Generalised) Linear Mixed-Effects Models in all analyses, and only response times for correct ‘no’ decisions were analysed. The Box-Cox test (Box & Cox, 1964) indicated that an inverse transformation was appropriate for the response-time data. Following Bates et al. (2018), the most parsimonious model for each analysis was selected by identifying the maximal random-effects structure (for the location parameter) that was supported by both the study design and the data, as determined through principal component analysis. In all models, log-likelihood ratio tests were used to determine whether the null hypothesis of no effect could be rejected by comparing a model containing the predictor of interest with a model from which the predictor had been removed.

*Analysis 1* tested for a morpheme interference effect by comparing accuracy and response times for nonwords with versus without morphological structure. Morphological structure was sum-contrast coded (0.5 for morphologically structured nonwords, − 0.5 for orthographic controls). Both accuracy and response time models included correlated varying intercepts and slopes for participants and varying intercepts for items.

*Analysis 2* examined which of the three definitions – dictionary-based type frequency, orthography-based type frequency, or orthography-based type frequency accounting for the false alarm penalty – best captured the participants’ performance on morphologically structured nonwords. For both accuracy and response-time data, three models were constructed for each (i.e., six models in total). In *Model 1*, the independent variable was the dictionary-based type frequency; in *Model 2*, the orthography-based type frequency was used as the predictor; and in *Model 3*, the false alarm penalty was included as a predictor in addition to the orthography-based type frequency. To reduce the skewness of the raw counts and normalise the distribution, the frequency variables in all models were log-transformed. All six models included varying intercepts for participants and items. Model selection relied on the Akaike Information Criterion (AIC; Akaike, 1973, 1974) and Bayesian Information Criterion (BIC; Schwarz, 1978), with smaller values indicating a better fit to the data.

## Results

### Analysis 1: The morpheme interference effect

**Fig. 1:**
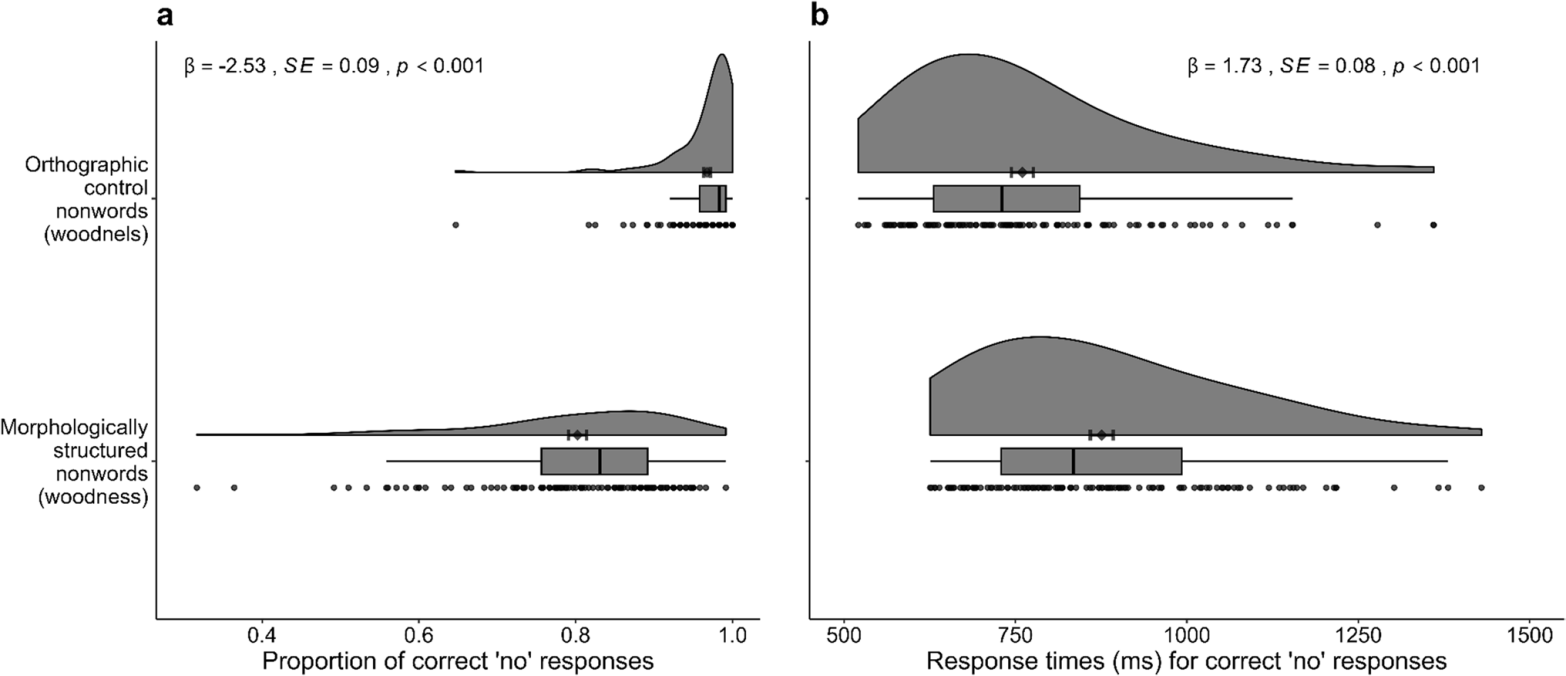
The morpheme interference effect on accuracy (**a**) and response time data (**b**). On each panel, the dots represent the average data for each participant. The box-and-whisker plots display the four quartiles of the ordered data, with the vertical lines inside the boxes indicating the medians (50%) and the edges of the boxes representing the lower and upper 25% quartiles. The whiskers show the expected variation in the data. The clouds illustrate the estimated data distribution, along with the means (represented by the dots) and one standard error of the mean (the error bars). Statistical analysis results are reported at the top of each panel

Figure 1 visualises accuracy (proportion of correct responses) and response times for correct ‘no’ responses to morphologically structured nonwords (e.g., *woodness*) and their orthographic controls (e.g., *woodnels*). Participants made *more* errors rejecting the morphologically structured nonwords (mean proportion correct = 0.80, *SD* = 0.13) than the control nonwords (mean proportion correct = 0.97, *SD* = 0.05; *ꞵ* = − 2.53, *SE* = 0.09, *z* = −28.5, *p* <.001). They were also *slower* at rejecting the morphologically structured nonwords (mean response time (RT) = 876 ms, SD = 183 ms) compared to nonwords without morphological structure (mean RT = 760 ms, *SD* = 175 ms; *ꞵ* = 1.73, *SE* = 0.08, *t* = 23.07, *p* <.001). Having established the morpheme interference effect in both accuracy and response times, we proceeded to Analysis 2, which examined whether responses to morphologically structured nonwords varied based on the properties of the affixes they contained.

### Analysis 2: Model selection for the nonword data

**Fig. 2:**
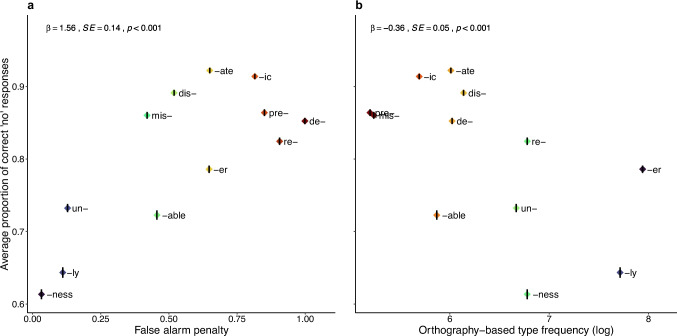
Average response accuracy for nonwords with each of the 12 affixes as a function of the false alarm penalty (**a**) and orthography-based type frequency (**b**). The coloured diamond shapes represent the mean proportion of correct ‘no’ responses for each affix, with the black vertical lines inside the diamonds indicating one standard error of the mean. Redder diamond colours represent ‘worse’ affixes (those with higher false alarm rates and lower frequency), while bluer colours represent ‘better’ affixes (those with fewer false alarms and higher frequency). The affix colours differ across panels because there is not always a direct correspondence between the number of distinct words in which an affix can be identified and its involvement in false alarms (e.g., the suffix *-ness* participates in fewer false alarms than *-ly*, but *-ly* is much more frequent). Statistical analysis results from the model incorporating both variables are reported at the top of each panel

*Accuracy.* The variables of interest – dictionary-based type frequency, orthography-based type frequency, and the combination of orthography-based type frequency and false alarm penalty – were tested in separate models (Models 1–3). The output of all three models, including their AIC and BIC values, is reported in Table 2. Each model yielded a significant result, indicating that each of the three definitions of morpheme experience accounted for some variance in participants’ performance. However, AIC and BIC values clearly indicate that the model incorporating both orthography-based type frequency and false alarm penalty (Model 3) provided the best fit for the data. This model shows that nonwords with affixes that appear in many distinct words (higher orthography-based type frequency) and participate in few false alarms (lower penalty) were more difficult to reject than those with affixes that occur in fewer words (lower orthography-based type frequency) and are involved in more false alarms (higher penalty). These effects are visualised in Fig. 2, which shows the average response accuracy for nonwords with each of the 12 affixes, plotted as a function of the affix’s orthography-based type frequency and false alarm penalty.

**Table 2:** Model comparison results for the accuracy data. Lower AIC and BIC values indicate a better model fit to the data

Model	Predictor	*ꞵ*	*SE*	*z*	*p*	AIC	BIC
1	Dictionary-based type frequency (log)	− 0.38	0.05	− 7.21	<.001	11,999	12,029
2	Orthography-based type frequency (log)	− 0.59	0.05	− 11.73	<.001	11,919	11,949
3	Orthography-based type frequency (log)	− 0.36	0.05	− 6.98	<.001	11,796	11,834
	False alarm penalty	1.56	0.14	11.47	<.001

**Fig. 3:**
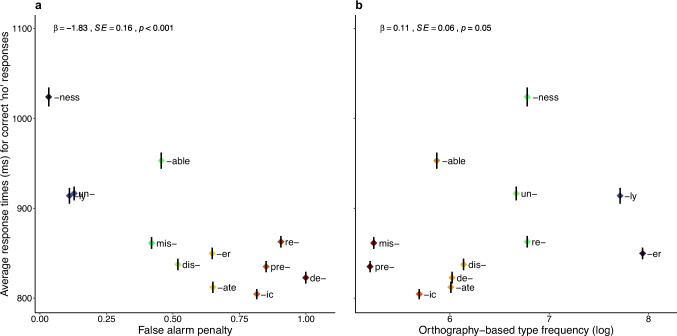
Average response times (ms) for nonwords with each of the 12 affixes as a function of the false alarm penalty (**a**) and orthography-based type frequency (**b**). The diamond shapes represent the mean response times (in milliseconds) for correct ‘no’ responses for each affix, with the black vertical lines inside the diamonds indicating one standard error of the mean. Like in Fig. 2, redder diamond colours represent ‘worse’ affixes (those with higher false alarm rates and lower frequency), while bluer colours represent ‘better’ affixes (those with fewer false alarms and higher frequency). Statistical analysis results from the model incorporating both variables are reported at the top of each panel

*Response times*. The results for the response-time data are presented in Table 3. Models that included only the frequency measures as predictors – dictionary-based type frequency in Model 1 and orthography-based type frequency in Model 2 – both returned significant effects, indicating that each of the two frequency measures, individually, accounted for some variability in response times for correct ‘no’ responses. However, when the orthography-based type frequency was combined with the false alarm penalty in Model 3, the frequency variable no longer explained any additional variance that had not already been accounted for by the false alarm penalty. These patterns are visualised in Fig. 3. The AIC and the BIC values indicated that Model 3 provided the best fit to the data. This result suggests that the speed with which readers in our sample rejected morphologically structured nonwords was best accounted for by the extent to which the affixes in these nonwords participate in false alarms. Specifically, nonwords containing affixes with a higher false alarm penalty were rejected more quickly than those with affixes with a lower false alarm penalty.

**Table 3:** Model comparison results for the response time data. Lower AIC and BIC values indicate a better model fit

Model	Predictor	*ꞵ*	*SE*	*t*	*p*	AIC	BIC
1	Dictionary-based type frequency (log)	0.18	0.06	3.21	.001	61,235	61,272
2	Orthography-based type frequency (log)	0.35	0.06	6.23	<.001	61,207	61,244
3	Orthography-based type frequency (log)	0.11	0.06	1.94	.05	61,078	61,122
	False alarm penalty	− 1.83	0.16	− 11.73	<.001

## Discussion

Morpheme knowledge is a vital component of skilled reading as it enables us to compute the meanings of both familiar and unfamiliar printed words (Castles et al., 2018; Rastle, 2019a). There is agreement that text experience plays a significant role in the development of morphological knowledge (Rastle, 2019b). Existing theoretical perspectives on morpheme learning argue that the way individual morphemes are presented in text is not uniform, and this variability likely influences how morphemes are learned (e.g., Plaut & Gonnerman, 2000; Ulicheva et al., 2020). A recent analysis of morphological information in a large collection of popular children’s books provided a concrete description of how this variability manifests in English. Korochkina and Rastle (2025) demonstrated that a large portion of morphologically complex words in English are spelled in ways that make identifying affixes difficult, which likely prevents them from being included in readers’ morpheme experience. They further showed that certain words are spelled in ways that may lead to incorrect morphological parsing and hypothesised that this could create confusion about the function of particular affixes and thus hinder learning (Korochkina & Rastle, 2025). The present study evaluated this perspective on morpheme learning in comparison to other possible formulations, using the morpheme interference paradigm to assess skilled readers’ affix knowledge.

We tested three possible definitions of what constitutes a reader’s experience with an affix: the first definition followed a linguistic perspective, in which a word is considered morphologically complex if it is formed through derivational affixation, regardless of whether the affix is easily identifiable (i.e., dictionary-based type frequency). The second definition included words that were likely to be *perceived* as complex by a reader *without* sophisticated etymological knowledge (e.g., orthography-based type frequency). This definition encompassed those genuinely complex words in which affixes were easily identifiable as well as words that merely *appeared* complex based on their spelling (i.e., false alarms). The third definition built on the second but posited that false alarms should incur a learning penalty (i.e., orthography-based type frequency adjusted for the false alarm penalty). We investigated which of these definitions better captured readers’ ability to reject morphologically structured nonwords comprising different affixes.

Our findings showed a robust morpheme interference effect on both accuracy and response times: participants made more errors and took longer to reject nonwords with morphological structure compared to those without. These effects replicate previous findings (e.g., Crepaldi et al., 2010; Dawson et al., 2018), and indicate that the adult readers in our sample had acquired sensitivity to morphological structure. The morpheme interference paradigm is commonly used in the literature to assess morpheme knowledge; however, we acknowledge that it does not allow for precise conclusions about the stage of lexical processing at which these effects occur. Future research could investigate this issue by combining a lexical decision task with EEG recording, which allows for more precise temporal resolution of such effects (see Lavric et al., 2012, for a possible design). Our analysis demonstrated that skilled readers’ processing of morphologically structured nonwords reflected distributional properties of particular affixes in text. Regardless of whether the affix type frequency was measured using a dictionary-based or orthography-based approach, participants made fewer errors and were quicker to reject nonwords with affixes that appeared with a small number of distinct stems. Importantly, the model using the orthography-based metric outperformed the dictionary-based model in terms of data fit. This result suggests that participants’ knowledge of affixes is *better* explained by distributional characteristics that arise from *orthographic* patterns, rather than by characteristics that reflect their true morphological status as listed in the dictionary. Critically, participants also found it easier to reject nonwords with affixes that were involved in many false alarms (and thus incurred a higher learning penalty), and the model incorporating both orthography-based type frequency and false alarm penalty provided the best data fit overall.

This result is in line with the theoretical account developed by Korochkina and Rastle (2025), and highlights the important role that the presentation of morphemes in written language plays in the acquisition of morpheme knowledge.^1^ This result also aligns with previous findings that morpheme knowledge is influenced not only by the amount of exposure to a morpheme but also by how reliably it conveys meaning (e.g., Hsieh et al., 2024). Exposure to certain affixes may seem substantial if we examine how frequently a given affix appears in distinct words historically formed by adding this affix to their stem. However, our research shows that if this structural complexity is not apparent to the reader, their experience with many of these words may have little impact on what and how they learn about the functions of the individual affixes. To illustrate, the prefix *de-* is encountered in 413 genuinely complex words in the 1,200 books popular with British children and adolescents that we used to extract information about individual affixes (Korochkina et al., 2024). However, readers are likely to identify this prefix in no more than half of these words (*N* = 214) because the other half contain bound stems that do not function independently in modern English, making affix identification far from straightforward (e.g., *demand*, *deceive*, *deduce*, *deplore*, *detain*). Moreover, learning of the prefix *de-* is likely to be undermined by the fact that there are a large number of false alarms (*N* = 199) involving this prefix. For instance, the word *debit* is unrelated to *bit* – ‘a small piece’, *decent* is unrelated to *cent* – ‘a unit of money’, *deliver* is unrelated to *liver* – ‘a large organ in the abdomen’, and *defeat* is unrelated to *feat* – ‘an achievement’. Because the number of false alarms involving *de-* is nearly equal to the number of useful, identifiable words with this prefix, uncertainty about the function of this letter cluster is high, as reflected in the learning penalty of 0.999. In contrast, the prefix *mis-* is much less common, but it is easily identifiable in the vast majority of the words in which it occurs (172/179), and it participates in only 16 false alarms, resulting in a much lower learning penalty (0.420). Expert readers in our study were slower to reject nonwords with *mis-* than with *de-* (Fig. 3), indicating acquired sensitivity to these idiosyncrasies in the presentation of the two prefixes in print.

In conclusion, our study represents a critical step toward a concrete and psychologically realistic understanding of how the acquisition of morpheme knowledge unfolds. Importantly, we believe that the significance of our work extends beyond a single experiment or topic. Languages differ in their morphological systems and orthographies, so the specific patterns observed in our study may not generalise beyond English. However, the finding that readers’ affix knowledge aligns with what is readily available to them through orthography underscores the limitations of theoretical approaches to language learning that rely on experience proxies *detached* from the perspective of typical readers, who are not linguists. This finding also highlights important avenues for future research into the role of words with bound stems in affix learning. Given their prevalence in English, repeated exposure to such words may contribute to affix familiarity by reinforcing recurring orthographic patterns (e.g., *sub-* in *submit, subsume; in-* in *include, induce*). Indeed, our linguistically informed definition (Model 1) accounted for some variance in participants’ data. However, because Model 1 included both words with bound stems and those with free-standing stems, it remains unclear to what extent the former contributed to this effect. Future research should therefore investigate how experience with words with opaque morphological structure supports the development of affix knowledge. Overall, our study provides a concrete example of how patterns observed in language corpora can be connected with those that characterise human language processing and thus contribute to the development of theories of learning through experience that are *psychologically* valid.

## Data Availability

All data and materials are available via the Open Science Framework (OSF) at https://osf.io/yq9h7/.

## Footnotes

1 We note that this finding does not imply that reading experience outweighs all other forms of input relevant to morpheme learning; rather, it indicates that specific features of English orthography are closely aligned with the patterns of morpheme knowledge observed in readers.

## Code availability

Analysis code is available via the Open Science Framework (OSF) at https://osf.io/yq9h7/.
